# Personalized Music-Embedded Sound Therapy Based on Gating Modulation and Neural Decoupling Reduces Tinnitus Severity

**DOI:** 10.3390/brainsci16060644

**Published:** 2026-06-17

**Authors:** Pablo I. Henriquez, Paul H. Delano, Javiera Herrada, Claudia Guevara, Hayo A. Breinbauer

**Affiliations:** 1 Department of Otolaryngology, Hospital Clínico Universidad de Chile, Santiago 8380456, Chile; pablohen8@gmail.com (P.I.H.); pdelano@hcuch.cl (P.H.D.);; 2 Department of Neurosciences, Facultad de Medicina, Universidad de Chile, Santiago 8380453, Chile; 3 Biomedical Neurosciences Institute (BNI), Facultad de Medicina, Universidad de Chile, Santiago 8380453, Chile; 4 Advanced Center for Electronical Engineer (AC3E), Valparaíso 2340000, Chile; 5 Department of Otolaryngology, Facultad de Medicina Clínica Alemana, Universidad del Desarrollo, Santiago 7610658, Chile

**Keywords:** tinnitus, sound therapy, coordinated reset, desynchronization, music-embedded therapy

## Abstract

**Highlights:**

**What are the main findings?**

• Personalized modified Music-Integrated Desynchronization Sound Therapy (mMIDST) produced significantly greater reductions in Tinnitus Handicap Inventory scores at 2 and 3 months compared with an control non-personalized low-frequency stimulation control.
• Clinical improvement was achieved with a short, practical daily stimulation schedule and was modulated by psychological comorbidity, with greater benefit in patients showing normal Goldberg scores.

**What are the implications of the main findings?**

• Embedding desynchronization-based tonal stimulation within music represents a feasible, well tolerated, and personalized non-invasive treatment strategy for chronic tinnitus.
• Future studies integrating electrophysiological biomarkers with clinical outcomes are required to clarify neural mechanisms and long-term therapeutic durability.

**Abstract:**

**Background:** Tinnitus is a prevalent auditory disorder associated with maladaptive cortical plasticity and aberrant neural synchronization across auditory and non-auditory brain networks. Acoustic desynchronization-based sound therapies, such as coordinated reset neuromodulation, aim to counteract pathological oscillatory patterns but commonly require prolonged daily listening sessions and specialized delivery formats, which may limit their accessibility and practicality in routine clinical settings. To address this limitation, a modified desynchronization protocol embedding therapeutic tones within music was developed to improve tolerability and engagement. This study aimed to evaluate the clinical effects of modified Music-Integrated Desynchronization Sound Therapy (mMIDST) on tinnitus severity in patients with chronic tinnitus. **Methods:** In this prospective, randomized, controlled, single-blind pilot trial conducted at the Otolaryngology Department of Hospital Clínico Universidad de Chile (Santiago, Chile) between July 2024 and July 2025, adults aged 18–75 years with chronic non-pulsatile tinnitus were assigned to receive either mMIDST or an active control intervention consisting of low-frequency stimulation (LFS) embedded within identical music tracks. Participants listened to personalized sound files for one hour daily, five days per week. Tinnitus severity was assessed using the Tinnitus Handicap Inventory (THI), with audiometric evaluations performed at baseline and after one, two, and three months. Between-group differences were analyzed using the Mann–Whitney U test. **Results:** Twenty-five participants completed the study (15 mMIDST, 10 LFS). Baseline audiometric thresholds and THI scores were comparable between groups. The mMIDST group showed significantly greater reductions in THI scores than the LFS group at two and three months of treatment (*p* < 0.05). **Conclusions:** mMIDST was associated with time-dependent improvements in tinnitus-related distress compared with an active control condition. Embedding desynchronization-based tonal stimulation within music may represent a promising and well-tolerated non-invasive approach for chronic tinnitus management.

## 1. Introduction

Tinnitus is characterized by the perception of sound without an external or internal auditory source. It can be classified as *objective tinnitus* when the perceived sound originates from a somatic source, such as blood flow or middle-ear muscle contractions, or as *subjective tinnitus* when the perceived noise has no somatic cause and therefore can be heard only by the patient. The latter form, understood as a *phantom auditory percept*, is more prevalent and constitutes the focus of this study. Current estimations suggest that tinnitus affects 5–20% of the population at some point in life [1,2], with its prevalence rising among older adults, affecting roughly 30–35% of people over 70 years old and reducing their quality of life by nearly 40% [3]. For most individuals, tinnitus remains a perceptual symptom, just the awareness of sound which has no identifiable source, with little or no impact on quality of life. However, in 1–3% of cases, it evolves into a distressing and burdening syndrome, which involves a plethora of uncomfortable reactions, including cognitive and emotional alterations such as anxiety, insomnia, and depressive symptoms, among others [3]. This distinction has become crucial for tinnitus research, underscoring the need to differentiate *sensory tinnitus* from *tinnitus disorder* with broader affective and cognitive comorbidities.

**Pathophysiological mechanisms. Increased central gain and beyond.** The underlying mechanisms of tinnitus, and particularly, the processes differentiating sensory tinnitus from tinnitus disorder, remain poorly understood [4]. Between 50% and 80% of tinnitus patients exhibit peripheral hearing loss due to inner-ear damage such as loss of cochlear hair cells secondary to acoustic trauma, ototoxicity, or presbycusis [3,5,6]. It has been proposed that the onset of tinnitus almost invariably involves at least minimal cochlear deafferentation, even in the form of “hidden hearing loss,” to which the central auditory system maladaptively responds [7]. The *central gain hypothesis* posits that decreased afferent input to the cochlea and auditory nerve triggers compensatory hyperactivity in central auditory structures, generating tinnitus perception [8,9], yet recent work suggests that increased central gain more accurately accounts for *hyperacusis*, an intolerance to loud sounds, rather than tinnitus itself [10].

**Network-level and gating models.** The heterogeneity of tinnitus phenotypes and the frequent presence of non-auditory symptoms (emotional, cognitive, attention-related, etc.), makes a single mechanistic explanation for all patients probably insufficient. Furthermore, tinnitus is increasingly viewed as a network-level disorder involving aberrant interactions among auditory, limbic, and frontostriatal regions. Supporting this view, older adults with sensory tinnitus show increased gray-matter volume in basal-ganglia nuclei, including the putamen, caudate, pallidum, and nucleus accumbens, without electrophysiological evidence of enhanced central gain [4]. These findings align with the *frontostriatal gating hypothesis* [11,12], suggesting that basal-ganglia circuits modulate the salience and persistence of phantom auditory percepts. This mechanism may contribute importantly to the difference between sensory tinnitus and tinnitus disorder [4]. Interestingly, this framework resonates with the therapeutic rationale behind Jastreboff’s tinnitus retraining therapy (TRT). Based on clinical observation, TRT employs a *mixing point*, which is the sound intensity where an external auditory or musical stimulus matches the perceived loudness of the tinnitus to facilitate habituation. By progressively decreasing the external stimulus while tinnitus perception diminishes, TRT aims to engage subcortical gating and filtering mechanisms that reduce the conscious salience of tinnitus. The use of music, rather than emotionally neutral sound, has been proposed to increase this gating effect, likely by recruiting affective and attention-driven mechanisms [13]. This conceptual parallel highlights that both network and gating models converge on the modulation of central auditory processing as a therapeutic pathway.

**Hypersynchrony activity in tinnitus.** Convergent evidence demonstrates that tinnitus is associated with widespread cortical network dysfunction characterized by abnormal oscillatory activity across multiple frequency bands. EEG and MEG studies consistently report an increase in slow-wave delta activity (1–4 Hz) in auditory and temporoparietal cortices, particularly in regions affected by sensory deafferentation, a pattern interpreted as a marker of cortical deprivation and thalamocortical dysrhythmia. This pathological delta activity is frequently accompanied by a concomitant reduction in alpha-band power, reflecting diminished inhibitory control within auditory networks, and by abnormal increases in gamma-band power and coherence in primary and secondary auditory cortices [14,15,16]. Within this framework, delta-band oscillations are thought to represent the core pathological substrate of tinnitus, providing a permissive environment for aberrant high-frequency synchronization. Gamma-band activity, in turn, has been shown to correlate with tinnitus loudness rather than conscious awareness, suggesting that early sensory cortices encode the intensity of the percept once the pathological network state is established [17,18]. Experimental manipulations that transiently reduce tinnitus intensity, such as residual inhibition, have been shown to induce selective decreases in delta-band power in temporal regions, further supporting a causal link between delta activity and the tinnitus percept [19,20]. Collectively, these findings portray tinnitus as a disorder of maladaptive oscillatory coordination, in which deafferentation-related delta activity disrupts normal thalamocortical dynamics, weakens alpha-mediated inhibition, and facilitates the emergence of focal gamma hypersynchrony. This gamma activity has been interpreted as a form of local cortical “kindling” centered on a deafferented frequency region within the auditory tonotopic map, where spike-timing-dependent plasticity mechanisms promote self-sustained hypersynchrony and contribute to tinnitus persistence [20].

**From pathological hypersynchrony to acoustic coordinated reset neuromodulation.** *Coordinated reset (CR) neuromodulation*, originally introduced by Tass [21], delivers brief, high-frequency pulse trains that induce postsynaptic plasticity and weaken synchronous synaptic events. When applied through auditory stimuli (*acoustic CR*), temporally patterned tones surrounding the tinnitus pitch aim to disrupt pathological synchrony and reestablish physiological oscillatory balance [14]. In early proof-of-concept studies, participants undergoing acoustic CR displayed significant reductions in tinnitus loudness and annoyance alongside electrophysiological normalization, namely increased alpha and decreased delta rhythms after stimulation [22]. These results suggested that CR neuromodulation could induce lasting plastic changes beyond the stimulation period. However, subsequent research has yielded inconsistent outcomes. Meta-analyses and systematic reviews emphasize that, while CR therapy is safe and conceptually appealing, robust clinical efficacy remains unconfirmed due to limited randomized controlled evidence, small sample sizes, and high interindividual variability [23]. Additionally, practical limitations have hindered broader implementation: protocols typically require several hours of daily exposure, and the isolated tonal stimuli are often perceived as unpleasant, negatively impacting adherence [24]. Consequently, strategies to improve tolerability and engagement are essential for advancing CR-based interventions.

**A modified approach: Integrating CR neuromodulation with musical stimulation.** To address these limitations, our group developed a modified acoustic CR paradigm that integrates the therapeutic logic of *coordinated reset* with the *gating and mixing principles* of Jastreboff’s TRT model. Specifically, we designed an in-house software platform developed by the authors (**mMIDST generator**, version 4.0) that embeds the characteristic CR tonal patterns within music selected by each patient using the musical track as a *“Trojan horse”* to deliver neuromodulatory tones seamlessly. Careful calibration ensured that the CR tone intensity matched the average loudness of the accompanying music, producing a blended auditory tailored experience that theoretically preserves the therapeutic desynchronization mechanism while enhancing comfort and ecological validity. During therapy, patients are instructed to listen at the individual *mixing point*, where the combined sound equals their perceived tinnitus intensity, thereby theoretically promoting both cortical desynchronization and subcortical gating/filtering engagement. In the present exploratory pilot randomized controlled trial, we aimed to evaluate the clinical effects of personalized Music-Integrated Desynchronization Sound Therapy (mMIDST) on tinnitus severity in adults with chronic tinnitus compared with an active control condition using low-frequency stimulation embedded within music. We hypothesized that patients receiving mMIDST would demonstrate greater reductions in tinnitus-related distress, as measured by the Tinnitus Handicap Inventory (THI), compared with the control group after three months of therapy.

## 2. Materials and Methods

The study was conducted at the Otolaryngology Department of the *Hospital Clínico Universidad de Chile*. We carried out a prospective, single-blind, randomized controlled pilot trial with an active control group designed to evaluate the efficacy of a novel sound therapy for chronic tinnitus.

**Figure 1:**
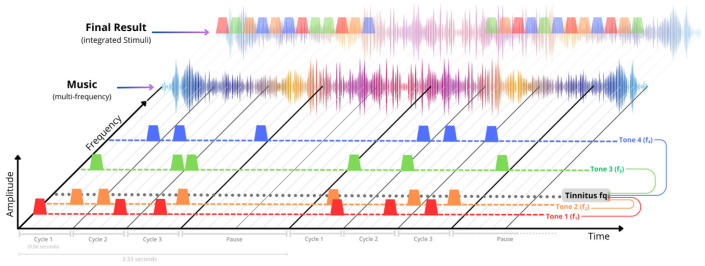
Stimulation cycles (Cycle 1, Cycle 2, and Cycle 3) are delivered, each followed by two pause cycles. Every stimulation cycle consists of four tones (f1, f2, f3, f4) presented in random order. The tones must be logarithmically equidistant from the patient’s tinnitus frequency (Ft). The full sequence of stimulation and pauses is administered continuously through auditory presentation for 1 h per day.

Both groups participated in a structured sound therapy program aimed at reducing tinnitus-related symptoms. Blinding was maintained at the participant level, as both interventions consisted of music-embedded auditory stimuli with similar perceptual characteristics, preventing subjects from identifying treatment allocation. The experimental group received modified Music-Integrated Desynchronization Sound Therapy (mMIDST), a personalized intervention developed to deliver temporally patterned tonal sequences tailored to each participant’s individual tinnitus frequency (Ft). Music tracks were freely selected by each participant from a curated set of classical music pieces according to personal preference prior to therapy initiation and served as the auditory carrier for the intervention. For each patient, the software generated four pure tones logarithmically distributed between 0.5 × Ft and 2.0 × Ft, embedded within the selected music tracks. This individualized configuration preserved the desynchronizing logic of coordinated reset neuromodulation while incorporating the mixing and gating principles of tinnitus retraining therapy (TRT) (Figure 1).

In contrast, the control group received a *low-frequency stimulation (LFS)* protocol, in which tones were randomly selected within a low-frequency range (500 to 2000 Hz), far from the spectral region typically associated with tinnitus perception in our patient population. These tones, also embedded within music, were matched in duration, intensity, and presentation structure but lacked the frequency-specific characteristics hypothesized to induce cortical desynchronization.

In this way, both groups (mMIDST and LFS) were exposed to sound therapies that were perceptually equivalent, including a similar music background, differing only in the temporal and tonal characteristics of the embedded tones that were tailored to induce desynchronization in the mMIDST group. This design ensured that the placebo condition remained acoustically and procedurally comparable to the active treatment, while omitting the theoretically critical temporal and frequency-specific component of the desynchronization protocol.

Participants were recruited by convenience sampling from the otolaryngology outpatient clinic. Inclusion criteria comprised adults aged 18 to 75 years presenting with unilateral or bilateral tonal chronic non-pulsatile tinnitus (>six months). Exclusion criteria included pulsatile or non-tonal tinnitus, a history of auditory hallucinations, Ménière’s disease, or any middle-ear pathology. Patients with severe hearing loss were excluded. Participants with normal hearing or mild-to-moderate hearing loss were matched across groups to ensure comparable auditory thresholds and minimize selection bias.

Participation was entirely voluntary. All participants received detailed information about the study procedures and provided written informed consent prior to enrollment.

The study protocol was reviewed and approved by the Ethics Committee of the Hospital Clínico Universidad de Chile (OAIC N° 1489/25)*,* in accordance with the principles of the Declaration of Helsinki.

### 2.1. Initial Assessment and Tinnitus Evaluation

At the first visit, all participants underwent a comprehensive otologic and clinical assessment to confirm study eligibility. This included a structured anamnesis exploring tinnitus duration, onset, and associated auditory or systemic symptoms, as well as relevant medical and pharmacological history. Each participant underwent otoscopic inspection followed by extended high-frequency audiometry covering frequencies from 0.125 to 16 kHz, using an Interacoustics^®^ AC40 audiometer (Interacoustics A/S, Middelfart, Denmark) with their own insert earphones under standard clinical conditions. Pure-tone average (PTA) was calculated as the mean air-conduction thresholds at 0.5, 1, 2, and 4 kHz (PTA4) for each ear. Hyperacusis was indexed as the number of audiometric frequencies with a detectable loudness discomfort level (LDL). Given the non-normal distribution of this variable, between-group comparisons were performed using the Mann–Whitney U test, while descriptive statistics are reported as mean ± standard deviation for consistency with other baseline variables.

### 2.2. Assessment of Tinnitus Severity and Psychological Comorbidity

Tinnitus severity and its impact on daily functioning were quantified using the Tinnitus Handicap Inventory (THI), the most widely validated questionnaire for assessing tinnitus-related distress. The THI comprises 25 items rated on a three-point Likert scale (“Yes”, “Sometimes”, “No”), grouped into functional, emotional, and catastrophic subscales. The total score provides a global index of perceived tinnitus handicap. Also, overall tinnitus severity and intensity was recorded by a Visual Analog Scale (VAS) for this percept. Psychological comorbidity was screened using the Goldberg Anxiety and Depression Scale (GADS), a brief and well-validated measure composed of two subscales (anxiety and depression), each containing nine dichotomous (Yes/No) items. Although not diagnostic, the GADS is sensitive to emotional distress and has been recommended for use in non-psychiatric populations, including tinnitus patients. In this study, it was used to characterize the affective and emotional dimensions potentially associated with tinnitus perception [25,26].

### 2.3. Tinnitus Pitch Determination

The individual tinnitus pitch was determined using a tinnitus-matching perceptual procedure. Initially, pairs of pure tones were sequentially presented between 250 and 10,000 Hz, at intensities ranging from 0 to 75 dB, and participants identified the tone that most closely matched their perceived tinnitus pitch. Subsequently, additional trials were conducted using pairs of tones with progressively smaller frequency differences centered around the initially selected pitch, allowing a fine-tuning of the match. This iterative process was repeated until the selected tinnitus pitch varied by less than 100 Hz across consecutive trials, at which point the measurement was considered reliable. Once the pitch was established, tinnitus loudness was matched using the same reference tone.

### 2.4. Study Design and Stimulation Protocols

Participants who met inclusion criteria were randomly assigned in a 1:1 ratio to either the modified Music-Integrated Desynchronization Sound Therapy (mMIDST) group or the low-frequency stimulation (LFS) control group using a computer-generated randomization sequence created prior to participant enrollment. Allocation concealment was ensured through sequential assignment performed by an investigator not involved in outcome assessment or data analysis. Due to the limited sample size and exploratory pilot design, no stratification procedures were applied. All participants received standardized instructions regarding listening environment, volume setting, and adherence. Participants were instructed to listen to the assigned tracks for 60 min/day, 5 days/week, for 12 weeks using their own headphones, in a quiet setting. Listening adherence was monitored using “last connection” in the digital folder interface, and participants were contacted every week to reinforce compliance and address technical issues.

### 2.5. Experimental Condition: mMIDST (Music-Integrated Desynchronization Sound Therapy)

The mMIDST protocol was designed to deliver a personalized form of acoustic desynchronization therapy, in which the tonal components were derived directly from each participant’s tinnitus frequency (Ft). For each subject, four pure tones were generated at logarithmically equidistant frequencies between 0.5 and 2.0 × Ft, approximating two octaves centered on the tinnitus pitch and covering the tonotopic region most likely involved in tinnitus-related cortical activity. The corresponding stimulation frequencies were

$$f_{1}=0.5Ft,f_{2}=0.79Ft,f_{3}=1.26Ft,f_{4}=2.0Ft$$

Music tracks were selected from a library of classical pieces. The library was curated to minimize abrupt dynamic changes and extreme spectral content that could interfere with tone perception and to ensure comparable musical structure across participants. Each participant selected the tracks from the same curated library, based on personal preference, which were then used as the acoustic carrier throughout the intervention. The embedded tones were presented at an intensity set relative to each participant’s tinnitus loudness match (see Section 2.3). The final audio files were exported in WAV/MP3, sampled at 48 kHz, and delivered via digital folder to the patient.

### 2.6. Temporal Structure of the Stimulation

In the mMIDST and LFS conditions, stimulation was delivered in repeating cycles consisting of an “ON” stimulation phase followed by an “OFF” pause phase. Each ON phase comprised 3 sequences of four tones (f_1_–f_4_), presented in random order, followed by an “OFF” pause phase. Specifically, each tone burst had a duration of 165 ms, including a rise/fall time of 10 ms ramps. A complete stimulation cycle consisted of 3 stimulation cycles of 0.66 seg each, followed by two pause cycles with the same duration, resulting in a total cycle length of 3 ON:2 OFF cycles repeated continuously throughout the daily 60 min session. To ensure consistent stimulation timing across participants, tone scheduling was generated algorithmically and exported as fixed audio files. The stimulation pattern (cycle structure, tone duration, ON:OFF pattern) was identical across participants; only the stimulation frequencies were individualized based on Ft.

### 2.7. Follow-Up Procedures

Adherence to the sound therapy protocol was monitored pragmatically throughout the intervention period. Participants were contacted weekly by telephone to reinforce usage instructions, address technical issues, and verify continued participation. In addition, upon completion of the three-month protocol, all participants were asked to complete a brief self-reported adherence questionnaire administered via an online digital platform, in which they confirmed regular use of the assigned sound therapy according to the prescribed schedule.

### 2.8. Statistical Analysis

Statistical analyses were performed using non-parametric methods due to the small sample size and the non-normal distribution of several clinical variables. Between-group comparisons were conducted using the Mann–Whitney U test, while within-group longitudinal changes were analyzed using the Wilcoxon signed-rank test. Statistical significance was defined as a two-tailed *p*-value < 0.05.

Given the exploratory pilot nature of this randomized controlled trial, no a priori sample size calculation was performed, and the sample size was determined pragmatically based on participant availability during the recruitment period. Accordingly, statistical analyses were intended to explore potential treatment effects and generate hypotheses for future adequately powered trials rather than to provide definitive efficacy estimates. No correction for multiple comparisons was applied, and results should therefore be interpreted cautiously within the context of an exploratory clinical study.

## 3. Results

A total of 25 patients were included in the analysis, with 15 assigned to the mMIDST group and 10 to the LFS group. Baseline demographic and clinical characteristics were comparable between groups (Table 1). The mean age was 59 years in the mMIDST group and 58 years in the LFS group. Tinnitus duration showed broad variability in both groups, ranging from 20 to 240 months in the mMIDST group and 10 to 240 months in the LFS group. The distribution of tinnitus laterality was similar, with the majority of patients in both groups reporting unilateral tinnitus.

**Figure 2:**
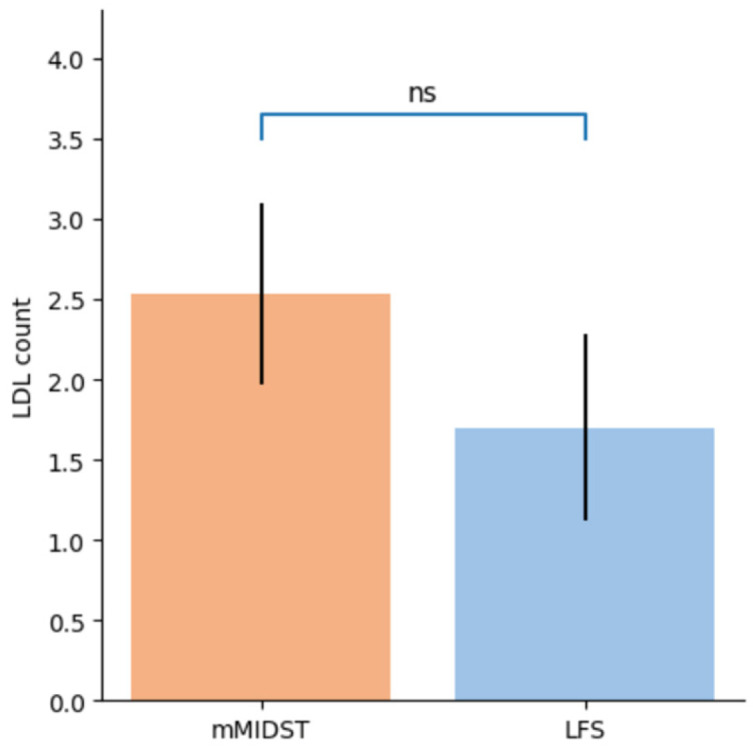
Mean loudness discomfort level (LDL) count is shown for the mMIDST and LFS groups at baseline. Bars represent mean values and error bars indicate the standard error of the mean (SEM). LDL count corresponds to the number of audiometric frequencies with a detectable loudness discomfort level per subject. No significant differences were observed between groups (Mann–Whitney U test, ns), indicating comparable degrees of sound intolerance prior to treatment.

Pure-tone audiometry results showed that both groups exhibited comparable degrees of hearing loss, with no significant differences in threshold levels across ears (Table 1). The mean PTA values were 33.5 ± 14.2 dB HL (left ear) and 32.1 ± 12.7 dB HL (right ear) in the mMIDST group, and 34.4 ± 16.0 dB HL (left ear) and 31.2 ± 12.8 dB HL (right ear) in the LFS group, with a similar distribution of hearing-loss severity categories. These values correspond predominantly to normal-to-mild hearing loss, with a minority of cases falling within the moderate range, and none within the severe range, consistent with the study’s exclusion criteria. Importantly, the distribution of hearing-loss severity categories (normal, mild, moderate) was similar between groups, indicating successful matching and reinforcing that the two cohorts were comparable in baseline auditory status. Mean LDL count did not differ between groups at baseline (mMIDST: 2.53 ± 2.2; LFS: 1.70 ± 1.8; *p* > 0.05), indicating a comparable degree of sound intolerance across treatment arms (Figure 2). Audiometric configurations were predominantly sloping across participants, consistent with high-frequency hearing loss commonly observed in tinnitus populations. No clear high-frequency notches suggestive of focal noise-induced patterns were systematically identified. The distribution of audiometric configurations was comparable between treatment groups at baseline.

Tinnitus pitch frequencies were homogeneously distributed across the study population and predominantly located within the high-frequency range. Across all participants, tinnitus pitch ranged from 2000 to 8000 Hz, with a median frequency of 4600 Hz and an interquartile range between 3500 and 6500 Hz for the mMIDST group; and 4000 to 5700 Hz for the LFS group. The distribution of tinnitus frequencies was comparable between the mMIDST and LFS groups, with no significant between-group differences, indicating adequate matching of tinnitus spectral characteristics at baseline. There were no significant differences between groups in the mean perceived tinnitus pitch (4600 Hz in both the mMIDST and LFS group).

The mean THI score was comparable between groups at baseline, with the mMIDST group starting at 69 points and the LFS group at 67 points, indicating similar initial tinnitus severity. At one month, both groups showed minimal change (mMIDST 70, LFS 72). By two months the mMIDST group demonstrated a reduction to 55 points, whereas the LFS group reached 68 points, resulting in a significant between-group difference (*p* = 0.032). This pattern continued over time. At three months, THI scores in the mMIDST group further decreased to 50 points, while the LFS group stabilized at 70 points, yielding a significant difference at three months of therapy (*p* < 0.001).

**Figure 3:**
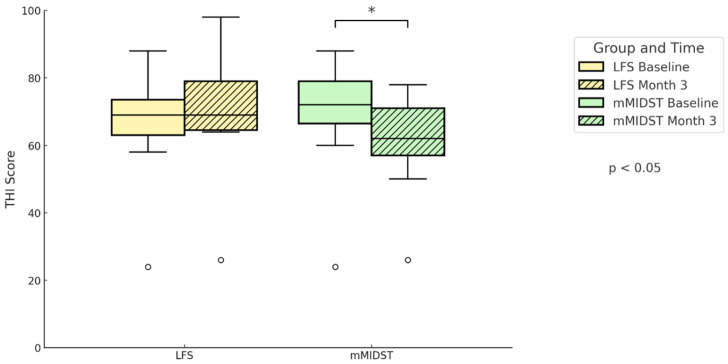
Grouped boxplots illustrating Tinnitus Handicap Inventory (THI) scores for the LFS and mMIDST groups at baseline and at the 3-month follow-up. Each treatment arm is represented by a pair of adjacent boxplots: solid fill indicates baseline values, whereas hatched boxes correspond to month 3. Small circles represent outliers. At baseline, both groups exhibited comparable THI distributions, with similar medians and interquartile ranges, indicating equivalent initial tinnitus severity. By month 3, the mMIDST group showed a downward shift in THI scores, reflected by a reduced median and decreased dispersion, whereas the LFS group demonstrated no appreciable change over time. Within-group comparisons over time were performed using the Wilcoxon signed-rank test, revealing a statistically significant reduction in THI scores in the mMIDST group (*p* < 0.05), as indicated by the significance bar and asterisk. No significant within-group change was observed in the LFS group.

**Figure 4:**
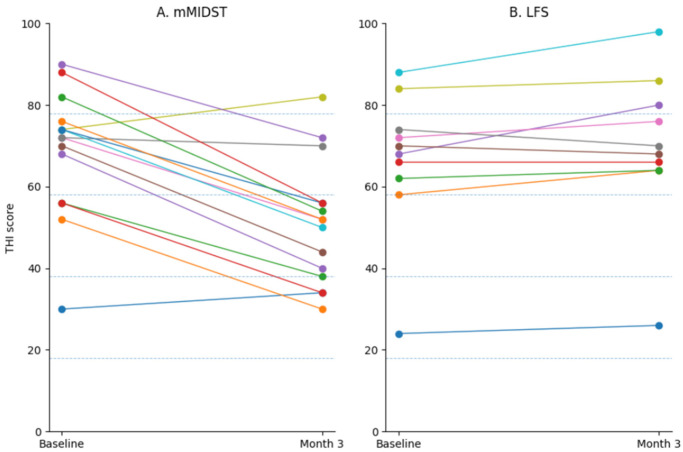
Individual changes in Tinnitus Handicap Inventory (THI) scores from baseline to 3 months are shown for both treatment groups. Each line represents a single subject, connecting baseline and 3-month assessments. (**A**) corresponds to the mMIDST group and (**B**) to the LFS group. THI scores are displayed on the *y*-axis in 20-point increments from 0 to 100. Horizontal dashed lines indicate standard THI severity categories (very mild, mild, moderate, severe, and catastrophic).

In terms of THI severity categories, both groups began with a predominance of moderate and severe tinnitus, with a smaller number of subjects in the catastrophic range. Over time, the distribution shifted in the mMIDST group: by month 2, several patients transitioned from severe to moderate or mild handicap, and by month 3 a subset reached the very mild category (THI ≤ 17). Conversely, the LFS group showed no comparable categorical shift; most subjects remained within their original severity levels, with some even transitioning from moderate to severe or catastrophic by month 3. This evolution mirrors the quantitative findings and aligns with the boxplot comparison (Figure 3), where the mMIDST group shows a downward displacement of the entire distribution and a statistically significant improvement, while the LFS group displays overlapping distributions with no significant change (Figure 4).

**Figure 5:**
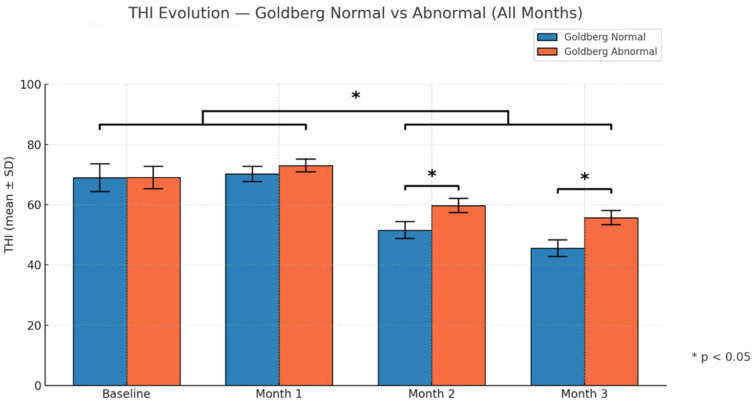
Evolution of Tinnitus Handicap Inventory (THI) scores across four monthly evaluations in patients from the mMIDST group, stratified by Goldberg test results (normal vs. abnormal). At baseline, both subgroups showed similar THI scores (mean = 69). At month 1, THI values remained comparable between subgroups. At months 2 and 3, lower mean THI scores were observed in patients with normal Goldberg results compared with those with abnormal results (month 2: 51 vs. 60; month 3: 46 vs. 56). Between-group comparisons at each time point were performed using the Mann–Whitney U test, showing statistically significant differences at month 2 and month 3 (*p* < 0.05), as indicated by the significance brackets. Error bars represent the standard deviation.

After the previous results, we stratified the mMIDST group by Goldberg test result (normal n = 9; abnormal n = 6). Participants with a normal Goldberg score maintained a stable THI at one month (69 → 70 points) and then showed decreases to 52 points at two months and 46 points at three months. Those with an abnormal Goldberg score exhibited a slight THI increase at one month (69 → 73 points) followed by reductions to 60 points at two months and 56 points at three months. The change between subgroups was significant at both two months (−8 points; *p* = 0.031) and three months (−10 points; *p* = 0.004), indicating a greater improvement in the subgroup with a normal Goldberg score (Figure 5).

## 4. Discussion

In this exploratory pilot–randomized controlled trial, we demonstrated that personalized music embedded desynchronization sound therapy reduced the THI score in patients with tinnitus disorder. The protocol, based on Tass’s desynchronization model and Jastreboff’s tinnitus retraining therapy principles, led to significant improvements in tinnitus loudness and annoyance compared with a sham condition using identical music containing non-therapeutic tones (LFS). Notably, these benefits were achieved after two months of therapy consisting of one hour of daily stimulation, which is considerably shorter than conventional CR regimens (4–6 h). Overall, this blended musical mMIDST tailored approach emerges as an efficient, well-tolerated, and patient-friendly intervention that maintains the theoretical integrity of mMIDST neuromodulation while enhancing practicality and adherence.

### 4.1. Mechanistic Considerations

Two main mechanisms may theoretically account for the observed clinical effects of this modified approach, based on the prior neurophysiological and neuromodulation literature.

First, embedding therapeutic tones within music enhances comfort and emotional engagement, addressing a key limitation of earlier studies, the repetitive and unpleasant nature of isolated tone sequences. Positive auditory contexts can activate reward and attention networks, promoting neuroplasticity and adherence. Second, the “mixing point” calibration, where external and internal sound intensities converge, enables subcortical gating of the tinnitus percept. As patients gradually reduce external sound levels, this process encourages adaptive filtering and habituation, akin to the logic of tinnitus retraining therapy. Thus, the therapy integrates two complementary mechanisms: mMIDST-induced network desynchronization and perceptual gating via auditory competition. It should be emphasized that these mechanistic interpretations are inferred from previous electrophysiological studies of coordinated reset neuromodulation and were not directly assessed in the present clinical trial.

These mechanisms also clarify discrepancies in the literature. Early studies linked coordinated acoustic reset to symptomatic improvement and EEG desynchronization (increased alpha, reduced delta/gamma activity) [22], whereas more recent trials found no significant effects. Systematic reviews continue to note methodological variability, small samples, and long daily usage times. Our results suggest that musical embedding and shorter exposure maintain CR mechanistic integrity while enhancing practicality and adherence.

### 4.2. Psychological Effects

An intriguing finding of this study was the differential response to mMIDST therapy depending on patients’ psychological profiles, as reflected by the Goldberg scale. Individuals with abnormal scores exhibited a less favorable improvement trajectory compared with those classified as normal, despite similar baseline tinnitus severity and auditory characteristics. This pattern suggests that emotional distress, anxiety, or depressive symptoms may interfere with the neural mechanisms underlying mMIDST-induced desynchronization and habituation. From an alternative and complementary perspective, it is also plausible that, in patients with abnormal Goldberg scores, the Tinnitus Handicap Inventory (THI) may capture a proportionally greater contribution of neuropsychiatric burden, such as anxiety, depressive symptoms, and emotional distress, rather than tinnitus perceptual severity per se. Under this framework, improvements driven primarily by auditory neuromodulation may be partially masked by persistent affective symptoms, resulting in a smaller apparent THI change despite potential modulation of the tinnitus signal itself. From a neurophysiological standpoint, heightened limbic or autonomic reactivity could sustain pathological coupling between auditory and non-auditory networks, thereby limiting the extent of neural plasticity achievable through acoustic stimulation alone. Clinically, these findings highlight the importance of integrating psychological assessment and support into tinnitus management, ensuring that interventions such as mMIDST are complemented by strategies addressing comorbid affective burden. Such a multimodal framework may optimize therapeutic outcomes, facilitate sustained engagement with treatment, and provide a more individualized approach to tinnitus rehabilitation. However, it should be noted that this subgroup analysis was exploratory, conducted post hoc, and involved relatively small subgroup sizes. Therefore, these findings should be interpreted cautiously and be considered hypothesis-generating. Further studies with larger cohorts specifically designed to evaluate psychological moderators of treatment response will be required before firm conclusions can be established.

### 4.3. Towards a Multidimensional Model of Tinnitus

Tinnitus is increasingly understood as a multifactorial brain disorder involving interconnected auditory, limbic, and attentional networks. Based on previous neurophysiological and neuroimaging studies, tinnitus has been associated with auditory cortical hypersynchrony, among several mechanisms proposed to sustain the symptom, in which impaired limbic and frontostriatal gating may fail to suppress aberrant auditory activity and disrupted thalamocortical rhythms, and deficient top-down modulation may reinforce tinnitus perception and its emotional salience [7,11,12,27]. Within this framework, psychological and neural processes interact bidirectionally: anxiety, hypervigilance, and depressive symptoms can heighten tinnitus awareness, while chronic tinnitus in turn exacerbates emotional distress [28]. Interventions capable of simultaneously engaging auditory and affective networks are therefore of particular interest. Music-based approaches are uniquely positioned in this regard, as they combine structured auditory input with emotional and attentional engagement, activating reward-related and gating networks while providing an ecologically valid carrier for desynchronizing stimulation. In this context, personalized and emotionally meaningful music may facilitate the effects of mMIDST therapy by enhancing tolerability, sustained engagement, and the interaction between sensory and non-sensory components of tinnitus processing.

Overall, tinnitus should be conceptualized as a dynamic, multi-level disorder encompassing peripheral, central, and affective domains. The present clinical findings are consistent with the hypothesis that addressing auditory-cortical hypersynchrony through individualized desynchronizing sound therapy may contribute to tinnitus relief, although direct neurophysiological confirmation was beyond the scope of this study. The present music-integrated mMIDST protocol can yield substantial relief when combined with complementary approaches targeting the remaining components of this complex condition. Importantly, while engagement of affective and attentional processes through music may facilitate tolerability and sustained use, the absence of significant improvement in the LFS control group underscores that modulation of auditory hypersynchrony remains a critical therapeutic component, accounting for the differential efficacy observed with mMIDST.

### 4.4. Limitations and Future Directions

Several limitations should be considered when interpreting the present findings. First, this study was conducted as an exploratory pilot randomized controlled trial with a relatively small sample size, which limits statistical power and restricts the generalizability of the results. No a priori sample size calculation was performed, and participant numbers were determined pragmatically based on recruitment feasibility; therefore, the findings should be interpreted as preliminary and hypothesis-generating rather than definitive estimates of treatment efficacy.

Second, although statistically significant differences in tinnitus-related distress were observed, confidence intervals and formal effect size estimates were not incorporated into the present exploratory analysis. Future adequately powered trials should include effect size reporting and precision estimates to better characterize the magnitude and clinical relevance of treatment effects.

Third, the follow-up period was limited to three months, preventing conclusions regarding the long-term durability and stability of therapeutic outcomes. Longer longitudinal studies will be required to determine whether clinical improvements are sustained over time.

Finally, although mechanistic interpretations were discussed in the context of neural desynchronization and gating models, no electrophysiological measurements (e.g., EEG biomarkers) were obtained in the present study. Therefore, proposed neural mechanisms remain theoretical and extrapolated from the prior literature. Future studies combining clinical outcomes with neurophysiological assessments will be necessary to determine whether mMIDST is associated with measurable changes in cortical oscillatory dynamics. Future studies integrating behavioral, audiological, and neurophysiological outcomes will be necessary to clarify the neural correlates underlying the observed clinical improvements.

## 5. Conclusions

mMIDST was associated with time-dependent improvements in tinnitus-related distress compared with an active control condition. Embedding desynchronization-based tonal stimulation within music may represent a feasible and patient-friendly neuromodulation strategy for chronic tinnitus management. Given the pilot nature of this study, these findings should be interpreted as preliminary and warrant confirmation in larger randomized trials.

This study demonstrates that a personalized music-integrated modification of acoustic coordinated reset neuromodulation, combining desynchronization principles with tinnitus gating and mixing-point frameworks, is associated with a significant reduction in tinnitus-related impairment as measured by the THI. Importantly, these clinical benefits were achieved within a short and practical daily listening schedule for two and three months of therapy, supporting the feasibility of this approach in real-world settings. Embedding therapeutic tonal sequences within balanced musical tracks preserved the core principles of desynchronizing coordinated acoustic reset while enhancing comfort, adherence, and emotional engagement. Within the limits of a clinically focused design, the present results support mMIDST as a personalized intervention capable of improving tinnitus-related quality of life. Future investigations combining behavioral outcomes with electrophysiological measures will be required to determine whether these clinical improvements are accompanied by corresponding changes in brain oscillatory activity and to evaluate the long-term durability of treatment effects across different tinnitus phenotypes.

## Figures and Tables

**Table 1:** Continuous variables were compared between groups using the Mann–Whitney U test. * Categorical variables (laterality and comorbidities) are presented descriptively and were not subjected to inferential statistical testing due to small cell counts. Values are expressed as mean (SD), median (IQR), or range, as appropriate. PTA was calculated as the mean air-conduction thresholds at 0.5, 1, 2, and 4 kHz (PTA4). THI = Tinnitus Handicap Inventory; LDL = loudness discomfort levels; dB HL = decibels hearing level; IQR = interquartile range.

	mMIDST Group (n = 15)	LFS Group (n = 10)	Statistical Comparison
Age, mean (range)	58 (48–65)	59 (54–62)	U = 75.5 (*p* = 1.00)
Tinnitus duration in months, median (IQR)	60 (39–90)	84 (39–138)	U = 65 (*p* = 0.59)
Laterality	Right: 5 Left: 8 Bilateral: 2	Right: 2 Left: 6 Bilateral: 2	- *
Comorbidities (n)	Hypertension: 4 Diabetes mellitus: 1 Hypothyroidism: 1	Hypertension: 2 Diabetes mellitus: 2 Hypothyroidism: 0	- *
Right ear PTA; mean (SD)	32.1 (±12.7) dB HL	31.2 (±12.8) dB HL	U = 92 (*p* = 0.35)
Left ear PTA; mean (SD)	33.5 (±14.2) dB HL	34.4 (±16.0) dB HL	U = 76.5 (*p* = 0.95)
THI score, mean (SD)	69 (±14.7)	67 (±17.5)	U = 85 (*p* = 0.59)
Tinnitus frequency, mean (SD)	4600 Hz (±1600 Hz)	4600 Hz (±1500 Hz)	U = 72.5 (*p* = 0.91)
LDL, count (SD)	2.53 (±2.2)	1.70 (±1.8)	U = 96 (*p* = 0.24)

## Data Availability

The raw data supporting the conclusions of this article are available at: https://github.com/HayoBK/mMIDST_Henriquez_2026_Data.

## Abbreviations

The following abbreviations are used in this manuscript:

mMIDST	modified Music-Integrated Desynchronization Sound Therapy
LFS	Low-Frequency Stimulation
CR	Coordinated reset
